# Evaluating a Novel Cat-Assisted Training (CAT) Intervention for Youth with Developmental Disabilities and Their Family Cat

**DOI:** 10.3390/ani16142133

**Published:** 2026-07-09

**Authors:** Delaney H. Frank, Saethra Darling, Kristen Moore, Kristyn R. Vitale, Megan MacDonald, Monique A. R. Udell

**Affiliations:** 1 Department of Animal and Rangeland Sciences, Oregon State University, Corvallis, OR 97331, USA; delaney.frank@oregonstate.edu (D.H.F.); saethra.darling@oregonstate.edu (S.D.); 2 4-H Youth Development, Oregon State University Extension Service, Beaverton, OR 97006, USA; kristen.moore@oregonstate.edu; 3 Cat Behavior Research Group, Maueyes Cat Science and Education, Marquette, MI 49855, USA; kristynrvitale@gmail.com; 4 College of Health, Oregon State University, Corvallis, OR 97331, USA; megan.macdonald@oregonstate.edu

**Keywords:** human–animal interaction, animal-assisted intervention, developmental disabilities, cat–child bond, domestic cat

## Abstract

Animal-assisted interventions (AAIs) for children with developmental disabilities (DD) have traditionally focused on dogs and horses, with less research on cat-centered AAIs. However, many potential AAI participants prefer, or only have access to, cats. The current Randomized Control Trial (RCT) study evaluated a novel Cat-Assisted Training (CAT) intervention designed to promote healthy behaviors in children, while also strengthening child–cat bonds. Participants included 36 children with DD (ages 8–17 years) and their family cats. Participating pairs were randomly assigned to either the CAT intervention (experimental) group or the waitlist control group. Feasibility was supported by 100% session attendance, a 94.4% post-intervention retention rate, and an 81% average homework completion rate. Participants in the CAT group reported increases in health-promoting activities, such as walking with their cat (*p* = 0.008), and demonstrated a significant increase in cat care responsibility (*p* = 0.039). Cats showed an immediate increase in sociability toward their child (*p* = 0.036), and a higher rate of secure attachment to their child partner at the one-year follow-up compared to baseline (*p* = 0.031). Overall, this initial study suggests that the CAT intervention represents a promising AAI model, providing empirical support that cats can be active and successful AAI partners for children with DD.

## Simple Summary

There has been a growing interest in animal-assisted interventions (AAIs) to support the wellbeing of children with developmental disabilities. While dogs and horses have commonly dominated AAI research, the potential for cats to serve as active AAI partners remains under-explored. The present study evaluated a novel Cat-Assisted Training (CAT) intervention for children aged 8–17 years with developmental disabilities and their family cats. Unlike previous work in which cats are often a passive presence, this program is among the first to involve cats as active intervention partners. Sessions were designed to support child health and development (joint physical activity and caregiving responsibility) and feline welfare through structured positive reinforcement reward-based training. Based on evaluation of outcomes, participation in the CAT intervention promoted healthy behaviors and strengthening of the bond between children with developmental disabilities and their family cats. The intervention also had a positive impact on cat social behavior. Overall, this study provides preliminary evidence that cats can serve as successful, active AAI partners for children with developmental disabilities while also potentially benefitting from improved human–cat relationships themselves.

## 1. Introduction

Animal-assisted interventions (AAIs) encompass a broad range of approaches that incorporate the use of animals to promote physical, psychological, and social wellbeing in people [1]. AAIs have gained increasing recognition as a promising approach to addressing the needs of children, including those with physical disabilities, learning disabilities, and social, emotional, or behavioral challenges [2,3]. Although there is preliminary evidence suggesting the potential benefits of AAIs incorporating multiple animal species, most of the research to date has focused on dogs and horses [4,5]. This is especially true for interventions where the animal is considered an active partner (e.g., animal training or walking together), in contrast to more passive roles such as simply being present or available for petting. However, not everyone who might benefit from AAI participation has access to dogs or horses, and some individuals may not feel comfortable interacting with these species. As AAI research continues to evolve, there is a need for more research on AAIs that include cats, to ensure equitable and accessible participation across diverse populations.

Domestic pet cats (*Felis catus*) are one of the world’s most popular companion animals, residing in over 49 million U.S. households, making them an important species to consider for AAI partnerships, especially when considering accessibility [6,7]. While cultural misconceptions often frame cats as aloof, cats, like dogs, are a facultatively social species [8]. Research indicates that cats possess many of the same traits that make dogs successful AAI partners including the ability to form attachment bonds with humans and robust trainability [9,10,11,12]. In addition, their smaller size, dexterity, and the fact that they often live in the home alongside us, rather than in separate housing, offer practical advantages for AAIs by facilitating more naturalistic, frequent opportunities for interaction [13]. However, incorporating cats into AAI settings also requires attentiveness to individual and species-specific behaviors and needs, including the potential for environmental sensitivities and neophobia. Therefore, despite evidence suggesting that AAIs centered on cat–human partnerships hold promise, there is still a need for carefully designed and monitored research studies to evaluate the efficacy and impact of cat-assisted interventions [14].

AAIs are also increasingly used to support children with developmental disabilities, offering structured interactions with animals to promote improved physical, cognitive, emotional, and social outcomes [15,16]. For youth with autism spectrum disorder and other neurodevelopmental conditions, AAIs have been associated with improved social participation, communication, emotional regulation, and engagement [17,18]. Systematic reviews and clinical studies indicate that children with DD derive significant health benefits from physical activity, including aerobic capacity and gross motor function, making interventions that embed movement into enjoyable contexts especially important [19,20]. AAIs can be a motivating vehicle for such activity. Beyond structured interventions, the human–animal bond with a companion pet can further enhance social skills, emotional wellbeing and, in some cases, physical activity in children with DD, by providing a calming, predictable partner that encourages interaction and routine [21,22]. Emerging work on cats suggests that many children with DD experience their family cat as a soothing, affectionate companion that reduces anxiety, supports prosocial behaviors, and offers an accessible source of comfort and attachment within the home environment [23,24]. Strengthening this existing child–cat bond through structured, positive interactions may therefore extend benefits beyond formal sessions, reinforcing both emotional and activity-related outcomes for the child long-term.

The welfare of the animal partner is also important to consider and evaluate when designing AAIs. There is increasing recognition that participating animals’ physical comfort, mental wellbeing, and agency are paramount to the success and ethical integrity of such interventions [25]. From a One Health perspective, AAIs should not prioritize human benefit at the expense of participating animals; instead animals should be provided with agency, specifically through choice and control over their engagement, and be monitored for indicators of stress [26]. Ideally, human and animal outcomes should be considered when evaluating new AAIs.

The current research focused on the development and evaluation of a novel cat-training animal-assisted intervention, CAT (Cat-Assisted Training), aimed at promoting healthy behaviors and improved relationships in children with developmental disabilities and their family cat. We also sought to design an AAI that would take child and cat autonomy and individuality into account, building flexibility into protocol progression and training activities based on individual skill level, daily progress, and also in response to the cat’s behavioral responses (for example monitoring indicators of stress, allowing choice to engage in a task or to take breaks). While taking such factors into consideration for human participants is not new, integrating similar responsiveness for animal partners can also support welfare by reducing stress and allowing greater control during interactions, which are key determinants of positive affective states in animals included in therapeutic and AAI activities [27].

Our primary aim was to investigate the outcomes of the CAT intervention on child and cat wellbeing. For children, we hypothesized that participating in the intervention would lead to improvements in health-related outcomes compared to baseline and when compared to a waitlist control group. Specifically we predicted that we would see an increase in responsibility taking behavior (engagement in cat-care activities), increased participation in joint activities, such as walking their cat at home and active training together (proxy measures for increased engagement and physical activity), improvements in empathetic treatment of animals, as well as the strength of the child’s attachment to their own cat. We also hypothesized that our One Health approach would help promote improved cat wellbeing and human–animal bond outcomes when compared to baseline measures and controls. Specifically, we predicted that cats participating in the intervention would show increased sociability with and attachment security to their child partner, both known proxies for stress reduction and increased stress resilience [28].

Given the limited number of randomized control studies investigating the efficacy of AAIs, especially those with cat partners, another aim of this study was to evaluate the acceptability and feasibility of implementing the CAT intervention in an applied setting. Based on the relative novelty of cat-training opportunities, coupled with prior research on cat-training and human–cat bonds [24] we hypothesized that participating children would be highly motivated to attend intervention sessions and actively engage in intervention activities. Specifically, we predicted that standard acceptability, fidelity, and engagement measurements would be at least as high for the CAT AAI as other successful interventions for this target population.

## 2. Materials and Methods

### 2.1. Participants

Participants were recruited from across the Pacific Northwest region of the United States through schools, community-based programs serving children with developmental disabilities, and through social media. Informed consent was obtained from the children’s guardians for both the child and the animal to participate in the study. Additionally, assent was obtained from the child participants after providing them with a clear explanation of the study’s purpose and procedures and allowing time to answer any questions.

To be eligible, children were required to be between the ages of 8 and 17 years old and have a developmental disability as reported by their parent or guardian. Additionally, they were required to own a household cat at the time of enrollment. The sample consisted of 36 children (55.6% male, 33.3% female, 2.8% non-binary, 8.3% declined to answer). The average age of participants was 13.0 years (SD = 3.14, range = 8–18). Most participants were White (72.2%), followed by Native American (11.1%), White/African American (5.6%), Latino or Hispanic (5.6%), White/Latino (2.8%), and African American or Black (2.8%). Prior to data collection, children underwent a brief standardized assessment to ensure their ability to follow simple directions [29].

A total of 36 pet cats were also included in this study. All cats were required to be at least 8 weeks of age and current on age-appropriate vaccinations, and for the safety of human participants and researchers, could not have a prior history of aggression towards humans. Demographic information was collected for 24 cats. Of the cats with a known sex and age, 58% (*n* = 14) were neutered males and 42% (*n* = 10) were spayed females. The average age was 3.5 years (SD = 3.76). Demographic data for the remaining 12 cats were unavailable due to non-response during retrospective data collection.

Upon enrollment and completion of baseline data collection, child–cat dyads were assigned to either the Cat-Assisted Training (CAT) intervention group (*n* = 18) or the waitlist control group (*n* = 18). Randomization was performed using a block randomization procedure to ensure equal group sizes throughout the recruitment period. The allocation sequence was generated by an independent statistician not involved in participant recruitment or data collection. Those assigned to the CAT group participated in assessments and the cat-training intervention described below. Participants assigned to the waitlist control group completed the assessments at matched timepoints with those in the CAT group and did not participate in the cat-training intervention during the study period. However, dyads assigned to waitlist control were offered the opportunity to participate in the cat-training classes after the completion of the study to provide them equal access to the opportunity.

### 2.2. Cat-Assisted Training (CAT) Intervention Design

The intervention comprised six 45 min sessions, held weekly or bi-weekly at dedicated research facilities. Each session was conducted in an animal-approved cat-proofed environment equipped with species-appropriate enrichment—including hiding spaces, scratching posts, and Classic Feliway^®^ diffusers (Ceva Santé Animale, Libourne, France)—to promote feline wellbeing during study activities. A researcher experienced in feline body language was present during all sessions to closely monitor and assess the cats for behavioral indicators of stress. If stress indicators were observed, mitigation strategies were implemented, such as adjusting environmental stimuli (e.g., dimming lights), providing supplemental hiding options, and offering the cat breaks from interaction. The presence of enrichment and the use of approaches that minimize stress and fear were also meant to facilitate discussions on these topics between researchers and child participants as part of the intervention. A consistent head trainer guided each dyad through a flexible cat-training and socialization curriculum (see Table S1 (Supplemental) for full set of lessons and behaviors associated with the training curriculum) tailored to the specific progress of the child and cat.

Sessions began by allowing the cat to acclimate to the space while the child discussed homework practice with the trainer/research team. The core curriculum focused on cat body language education, appropriate interaction techniques, and positive reinforcement reward-based training. Weekly homework targeted practical skills, such as practicing training mechanics, identifying their cat’s toy and treat preferences, and teaching specific behaviors such as sit, come, and target. These activities were specifically designed to foster the child’s involvement in their cat’s care and improve communicative clarity within the dyad.

### 2.3. Data Collection

Data were collected at three assessment timepoints for all participants: (T1) baseline prior to intervention, (T2) six weeks after baseline (post-intervention for the CAT group), and (T3) one year after baseline. During assessments, all children completed three validated self-report questionnaires:

1. Lexington Attachment to Pets Scale (LAPS) [30]

The LAPS is a 23-item instrument designed to measure the strength of children’s attachment to their pet. It asks participants to rate their agreement with statements about their relationship with and feelings towards their pet, ranging from 0 (disagree strongly) to 3 (total agreement) on a Likert scale. The LAPS can be categorized into three subscales: General Attachment (GA), Animal Rights and Welfare (AR), and Person Substituting (PS) [31]. GA includes statements relating to the general relationship the child has with their cat, such as “My cat and I have a very close relationship”. AR indicates the pet’s status in the household and includes statements such as “I think my cat is just a cat”. PS indicates how central the cat is to the participant’s life, and includes statements such as, “My cat means more to me than any of my friends”. Scores were summed across the three subscales to calculate a total LAPS score. We also evaluated responses to the GA subscale as a second measure, to evaluate the effect of the intervention on feelings of attachment alone (independent of general feelings about animal welfare or animal bonds as a possible substitute for human bonds). In both cases, higher scores indicated stronger feelings of attachment. The possible range of the LAPS’s total score was 0 to 46 and the GA range was 0 to 22.

2. Cat Care Responsibility Inventory (CCRI) [32]

The CCRI is an 18-item self-report survey designed to assess children’s responsibility for cat-related chores. Adapted from the Dog Care Responsibility Inventory, the CCRI asks children to indicate who among their household members (e.g., “me”, “mom”, “dad”, “bro/sis”) participates in each of the pet-care activities (e.g., “Usually brushes cat”, “Usually fixes cat’s meals”, “Usually plays with cat”). Household members are adapted to reflect the specific composition of each child’s household. For each item, children select all applicable household members who participate in the activity. Three items were excluded from the analyses because they measured activities outside of the scope of the intervention, and therefore no impact was expected for these activities: “Usually gives cat medicine”, “Usually goes to the vet with cat”, and “Usually washes cat”. Raw data were then converted into binary form: 0 if the child is not involved in the cat care activity and 1 if the child is involved. CCRI scores were then calculated by summing the number of activities in which the child participates across the 15 items, with a possible range of 0 to 15.

3. Children’s Treatment of Animals Questionnaire (CTAQ) [33]

The CTAQ is a 13-item self-report survey designed to measure children’s humane behavior towards their pet. Each item presents a scenario related to interacting with their pet cat (e.g., “Play with cat,” “Cuddle cat,” “Talk to cat”). Children are asked to indicate the frequency with which they engage in each behavior, using a three-point Likert scale of “Often,” “Sometimes,” or “Never.” CTAQ scores are calculated using the scoring method outlined in Thompson and Gullone’s (2003) [33] study. Higher scores indicated more frequent engagement in humane behaviors towards their pet cat, with a possible range of 13 to 39.

During all assessments, cats also participated in two behavioral evaluations with their child partner:

1. Cat Attachment to Child—Secure Base Test

**Figure 1:**
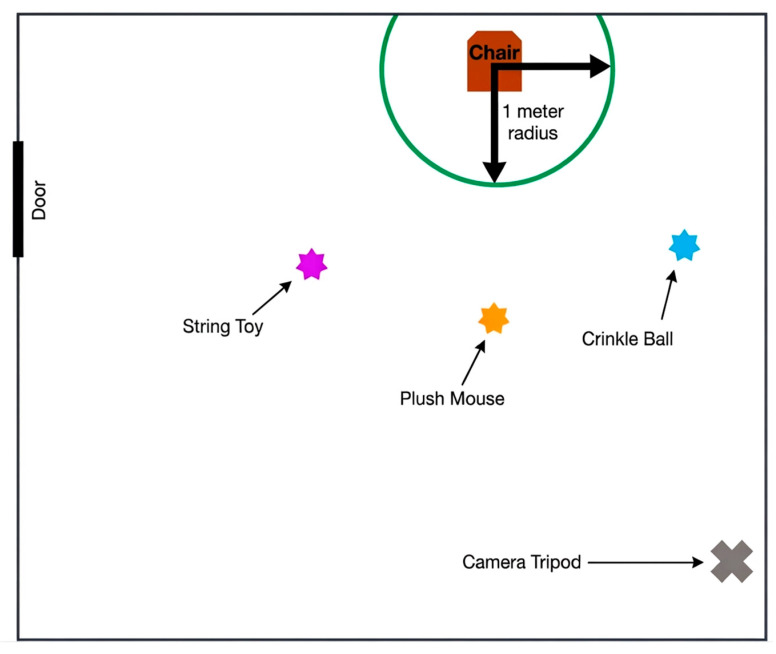
Secure Base Test layout. (Source: Authors’ own research). This test took place in a mostly empty room used for cat-behavior assessment. Three cat toys were present during testing. When present, the caregiver sat in the chair located within the 1 m-radius semi-circle.

Once the surveys were completed, the assessment transitioned to the Secure Base Test (SBT), a behavioral test used to evaluate cat attachment security towards their human partner. This behavioral assessment was conducted in a behavior testing room roughly 47 square meters that was unfamiliar to the cat at the start of the study. A chair was placed in a 1 m radius semi-circle for the human participant to sit in when present (Figure 1). Three cat toys were placed in the room and were present during all phases of the test: a string toy, a plush mouse, and a crinkle ball. All sessions were videotaped so behavior could be coded later by two independent research assistants.

The full assessment followed the standard SBT protocol [10] and lasted eight minutes, broken into four 2 min phases:

Phase 1 (Baseline): The child sat neutrally on the chair for two minutes while the cat was free to explore the room or interact with the child. If the cat chose to interact with the child, defined as coming inside the 1 m radius of the chair, the child could speak to and pet the cat. If the cat brought a toy into the semi-circle, the child could play with the cat. Otherwise, the child remained passive and quiet.

Phase 2 (Separation): The child exited the room, and the cat was alone for two minutes.

Phase 3 (Reunion): The child returned, greeted the cat if the cat was at the door, sat back on the chair and was instructed to behave as they did in Phase 1 for two minutes. The cat was allowed to behave freely.

Phase 4 (Active Interaction): The child remained in the chair but actively attempted to get the cat’s attention. They were told that they could call the cat and encourage them to stay nearby but could not leave the chair or use any physical restraint.

Final attachment classifications were determined through consensus coding of the SBT video recordings, following individual coding and inter-rater reliability (IRR) assessment [10,28]. Two independent coders evaluated the video footage from the end of the alone phase through the end of the return phase of the SBT using an ethogram (Table 1).

2. Cat Sociability Towards Child–Sociability Assessment

Sociability was quantified through the standardized coding of video footage from Phase 1 (cat-initiated sociability) and Phase 4 (child-initiated sociability) of the SBT. To ensure objective measurement, two independent coders evaluated the duration of proximity-seeking behavior (operationally defined as the cat having at least one paw within the taped semi-circle encompassing a 1 m radius around the child). Coders utilized continuous duration recording to capture the total time spent within this zone during each phase. To ensure consistency between the two independent coders, inter-rater reliability was calculated using an Intraclass Correlation Coefficient ICC(2,1). Reliability was excellent (ICC = 0.996), demonstrating high absolute agreement between the raters.

We also collected data on AAI efficacy throughout the intervention for participants assigned to the CAT group:

Attendance and Retention

Participant attendance was recorded for each of the six intervention sessions. The attendance rate was calculated as the percentage of sessions attended by the intervention group out of the total number of sessions. The retention rate was measured as the percentage of participants who completed the full study (remained enrolled and participated in data collection through T3) out of all enrolled participants. High acceptability in AAI studies is typically evidenced by attendance and retention rates exceeding 90% [29,30]. Previous cat and dog training interventions have reported rates between 86% and 91% [10,34], however it was unknown if this level of attendance and retention would apply to an intervention involving children with developmental disabilities and their household cats. We hypothesized, however, that this intervention would be feasible as designed and that the novelty of a cat-training intervention could further increase motivation to participate, resulting in attendance and retention rates equal to or greater than prior studies.

Homework Completion

At the beginning of each session, participants verbally self-reported completion of optional homework assignments. In prior studies, homework completion rates have served as a secondary measure of AAI acceptability as well as a measure of engagement, indicating a participant’s willingness and ability to practice and apply what they are learning in the program beyond the intervention setting. Reported completion rates across these studies have varied, for example a 64.96% completion rate was found in an intervention designed for middle schoolers with ADHD [35], while an 83% completion rate was reported for college students with ASD [36]. Given the age range of the current study, the tailored and unique nature of this intervention, and the involvement of a family cat, we predicted that the homework completion rate for this study should fall somewhere between these values (or higher).

Fidelity Data

Fidelity, or program adherence, refers to the extent to which program components were delivered exactly as prescribed [37]. One goal of measuring fidelity in the current study was to assess the consistency of program implementation across participants and to explore the relationship between fidelity and cat-training success. While 100% protocol adherence within applied settings (both interventions and animal training) is typically considered unrealistic, fidelity levels around 60% have previously been associated with success [38]. The current study utilized objective behavioral observations of recorded sessions to measure adherence to the intervention protocol. Balancing protocol adherence with necessary adaptations to allow the program to accommodate individual needs was also a priority. We predicted this approach would facilitate participant retention, engagement and primary health and wellbeing outcomes. Comparisons of which aspects of the program could be delivered in a highly consistent way across participants, and those that required greater adaptability, were calculated to provide information that could be helpful when designing and conducting future cat-training AAIs. To assess fidelity, two researchers reviewed recordings of each intervention session alongside each session’s progress logs. From these data, the actual implementation of the program could be compared with the protocol instructions for the prescribed core components outlined in Table S1 (Supplementary).

Cat-Training Progress

Cat-training progress was measured by logging progress across six possible targeted behaviors included in the CAT intervention: target training, recall, name attention, eye contact, sit, and go to mat. Session videos were reviewed to track the progress of each cat in these six behaviors. For each behavior, the cat’s training stage was assessed using a four-point scale:

Stage 1: Cat follows food in hand to perform behavior (luring).

Stage 2: Cat follows a hand signal or the target stick to perform behavior.

Stage 3: Cat starts learning a spoken word cue, but still relies on a visual cue (hand signal or use of target stick) to perform behavior.

Stage 4: Cat follows spoken word cue without assistance from a visual cue.

Due to the foundational nature of target training, this behavior was considered complete at Stage 2. All other behaviors were considered complete when the cat reached Stage 4 (trained behavior with full stimulus control). This represents a high standard of proficiency, as establishing reliable stimulus control typically requires precise reinforcement and repetitive practice that exceeds the scope of many standard, short-term introductory training classes.

### 2.4. Data Analysis

The primary outcomes of interest in this study focused on evaluation of the CAT AAI investigating child and cat behavior and wellbeing measures. Child-related outcomes included child-reported cat caregiving responsibility (CCRI), child-reported attachment strength (LAPS total score), and joint activity measures (walking and training behaviors). The primary measure of interest for the cat was cat sociability (specifically evaluating if the intervention led to significant increases in proximity-seeking behavior towards their child partner compared to controls—a variable associated with comfort level and preference) and cat attachment security to their child partner (a measure of caregiver facilitated stress-resilience as assessed by the Secure Base Test (SBT)). Additional exploratory analyses were conducted to further characterize the child–cat relationship at baseline (e.g., LAPS General Attachment), child-reported humane behavior toward the cat (CTAQ) and correlational analyses between behavioral and relationship variables. Implementation and feasibility data for the intervention (i.e., attendance, retention, and homework completion) were also evaluated.

All statistical analyses were conducted using R (version 4.5.1). All analyses utilized a complete-case approach, meaning participants were included in a specific analysis only if they provided complete data for all variables relevant to that measure at the designated timepoints. Initial descriptive statistics (means, standard deviations, and frequencies) were calculated to summarize participant demographics and the characteristics of the study variables. A baseline alpha level of α = 0.05 was utilized for all statistical tests. For our primary outcome measures, results are reported with their associated *p*-values. Exploratory and post hoc analyses were conducted to provide more information about the population, to evaluate possible explanations and to serve as a potential basis for future hypothesis-generation, including baseline correlations/comparisons, specific item-level measures, and subgroup assessments. Adjustments for multiple comparisons were not applied to exploratory measures and therefore such outcomes are treated with appropriate caution in the discussion.

#### 2.4.1. Baseline Child–Cat Interactions

Child–cat attachment, child caretaking responsibility for their cat, and humane behavior towards the cat were evaluated for all participants at baseline. Prior to inferential analyses, the assumption of normality for all continuous variables was assessed using the Shapiro–Wilk test. When both variables in a correlation-based comparison were confirmed to be normally distributed, Pearson’s product-moment correlation tests were conducted. This applied to comparisons involving the total LAPS score, and the scores from the CTAQ and CCRI inventories.

#### 2.4.2. Child Intervention Outcomes

To evaluate the impact of the CAT AAI on the child’s relationship with and care for their cat, data were analyzed from the CTAQ, LAPS, and the CCRI, including specific joint activity measures regarding teaching and walking their cat. The Shapiro–Wilk test was first used to confirm that the change scores for the surveys were normally distributed. Independent sample *t*-tests were then conducted to compare change scores (T2−T1) between the intervention (CAT) and control groups. Additionally, Fisher’s Exact Tests were employed to assess pre- to post-intervention changes in specific behavioral categories, such as the frequency of participants training or walking their cats.

#### 2.4.3. Cat Intervention Outcomes

Each cat was individually categorized into one of four attachment styles towards the child partner: Secure, Insecure-Avoidant, Insecure-Ambivalent, or Insecure-Disorganized. IRR was established on the initial independent classifications using Cohen’s kappa (ϰ ranged from 0.625 to 0.822 across timepoints), indicating substantial to almost perfect agreement. Following independent IRR calculation, the two coders came together to decide on the final classifications for any cats that they had not independently assigned to the same category in the first phase of classification. If agreement about category placement had not been reached, the cat would have been labeled unclassified as defined in the ethogram and would not have been included in the analysis. This did not apply to any cats.

To evaluate cat attachment towards the child from the SBT, attachment style classifications were dichotomized into Secure vs. Insecure (encompassing Avoidant, Ambivalent, and Disorganized). This allowed for the assessment of improvement status, defined as a longitudinal transition from an Insecure classification at baseline (T1) to a Secure classification at subsequent timepoints (T2 or T3). To evaluate the significance of these shifts in each group, McNemar’s tests were conducted to compare the proportion of secure to insecure classifications between T1 and the six-week follow-up T2, and T1 and the one-year follow-up T3.

To evaluate the impact of intervention participation on cat sociability, Wilcoxon Signed-Rank tests were conducted to compare the duration of proximity-seeking during both Phase 1 (cat-initiated sociability) and Phase 4 (child-initiated sociability) at baseline (T1) to subsequent timepoints (T2 and T3). Because the intervention was specifically structured to improve the cat–child bond, directional (one-tailed) tests were used to assess the expected increase in cat sociability toward the child.

#### 2.4.4. Intervention Feasibility

Attendance and Retention

Based on prior research, a program attendance rate of at least 90% was considered indicative of high intervention acceptability [34,38]. The retention rate was measured as the percentage of participants who completed T3 out of all enrolled participants. This rate was compared to the retention rates reported in similar dog studies to determine the acceptability of the intervention’s retention. Homework completion rates were calculated as the percentage of completed assignments out of all prescribed assignments.

Fidelity and Cat-Training Progress

To assess fidelity, two researchers reviewed recordings of sessions and session progress logs. They compared the actual implementation of the program to the prescribed core components outlined in Table S1. For each participant, the proportion of sessions that adhered to the protocol was calculated to obtain a fidelity percentage. For cat-training progress, the proportion of completed behaviors was calculated by dividing the number of completed behaviors by the total number of attempted behaviors. Spearman’s Rank correlation analysis was conducted to examine the relationship between program fidelity and behavior completion.

## 3. Results

### 3.1. Baseline Child–Cat Interaction Data

#### 3.1.1. Baseline Child Attachment and Care for Cat

A summary of data from baseline survey measures can be found in Table 2. Total LAPS scores reflected generally moderate to high levels of overall child–cat attachment; General Attachment (GA) sub-scores (the measure specifically focused on the child’s feelings of attachment towards their cat) were consistently high. Children also typically reported frequent humane behaviors (CTAQ) towards their cats. However, cat care responsibility (CCRI) scores varied more widely, reflecting diverse levels of child involvement in cat care.

#### 3.1.2. Baseline Comparability Between Groups

Baseline comparability was assessed for all primary demographic variables and survey metrics. As shown in Table 3, no statistically significant differences were observed between groups (all *p* > 0.05).

#### 3.1.3. Baseline Cat Attachment to Child

In total, 34 cat–child attachment dyads were classified into an attachment style at timepoint 1 (of the original 36 dyads one cat was unclassifiable and, due to a technical issue, the SBT video for one cat was lost). Of the classifiable cats, 29.4% (*n* = 10) were categorized as securely attached to the child participant and 70.6% (*n* = 24) were categorized as insecurely attached. Of the insecurely attached cats, 8.3% (*n* = 2) were ambivalent, 91.7% (*n* = 22) avoidant, and none were disorganized. When comparing 18 intervention cats and 16 classifiable control cats, there were no significant differences in the number of cats classified as Secure vs Insecure at baseline (Fisher’s Exact Test, *p* = 1.00, ϕ = −0.03).

#### 3.1.4. Baseline Relationship Between Reported Attachment and Reported Behavior Towards Cat

Pearson’s correlation tests were conducted between child responsibility for cat care (CCRI) and cat attachment (Total LAPS and GA subscale). A statistically significant positive correlation was observed between the child’s involvement in care and responsibility taking for their cat (CCRI) and reported levels of child attachment towards their cat as measured by the total LAPS score (*r* = 0.55, *p* = 0.00081, 95% CI [0.26, 0.75]) and their GA score (*r* = 0.60, *p* = 0.00019).

Pearson’s correlation tests were also computed between children’s humane behaviors towards their cat (CTAQ) and their cat attachment (Total LAPS and GA subscale). A statistically significant positive correlation was observed between CTAQ scores and GA scores (*r* = 0.46, *p* = 0.01) but not between CTAQ and Total LAPS scores (*r* = 0.17, *p* = 0.32).

### 3.2. Child Intervention Outcomes

#### 3.2.1. Children’s Treatment of Animals Questionnaire (CTAQ)

A total of 29 participants provided complete data for this survey at both timepoints and were included in the analysis. The Shapiro–Wilk test suggested that the CTAQ change scores (score at T2−score at T1) were normally distributed for both the CAT (*W* = 0.98, *p* = 0.93) and control (*W* = 0.91, *p* = 0.18) groups. To determine the effects of the intervention, an independent samples *t*-test was conducted on the change scores from T1 to T2 for the experimental and control groups. While child participants in the CAT group showed a slight mean increase in humane treatment scores post-intervention (*M* = 0.44) and the control group showed a slight mean decrease in these scores during the same period (*M* = −0.54), there was not a statistically significant difference in the changes observed between groups (*t*(27)= 1.02, *p* = 0.32, 95% CI [−0.985, 2937], Cohen’s *d* = 0.38).

#### 3.2.2. Lexington Attachment to Pets Scale (LAPS)

A total of 33 participants completed this survey at both timepoints and therefore were included in the analysis of LAPS data. The Shapiro–Wilk test indicated that LAPS change scores (LAPS score at T2−LAPS score at T1) followed a normal distribution for both the CAT (*W* = 0.89, *p* = 0.051) and control (*W* = 0.90, *p* = 0.085) groups. To determine the effects of the intervention on child-reported attachment towards their cat, an independent samples *t*-test was conducted on the change in LAPS scores from T1 to T2. Children in the CAT group showed a mean increase in reported attachment (with a mean increase in total LAPS score of 0.02) and the control group showed a mean decrease in reported attachment (with a mean decrease in LAPS score of 0.01); however, the difference observed between groups was not statistically significant (*t*(31) = 0.50, *p* = 0.62, 95% CI [−0.094, 0.155], Cohen’s *d* = 0.17).

#### 3.2.3. Cat Care Responsibility Inventory

A total of 29 participants provided complete data for the CCRI analysis. The Shapiro–Wilk test confirmed that CCRI change scores (CCRI score at T2−CCRI score at T1) followed a normal distribution for both CAT (*W* = 0.92, *p* = 0.11) and control (*W* = 0.91, *p* = 0.21) groups. Children in the CAT group demonstrated a mean increase in CCRI score of 1.56, suggesting increased involvement in cat care and responsibility taking post-intervention. The control group reported a mean decrease of 0.91. An independent samples *t*-test revealed a statistically significant difference in CCRI change scores between the groups (*t*(27) = 2.17, *p* = 0.039, 95% CI [0.136, 4.793], Cohen’s *d* = 0.83).

#### 3.2.4. Joint Activity Measures

Results from Fisher’s Exact Test on question 15 of the CCRI (“Teach cat new things”) revealed a statistically significant increase in the number of participants in the intervention group who reported teaching their cat new things, rising from 44.4% (*n* = 8) pre-intervention to 94.4% (*n* = 17) post-intervention (*p* = 0.0027, ϕ = 0.54, 95% CI [0.005, 0.433]). Similarly, for question 16 (“Walk cat”), the proportion of intervention participants who reported walking their cat increased from 27.8% (*n* = 5) pre-intervention to 77.8% (*n* = 14) post-intervention (*p* = 0.008, ϕ = 0.50, 95% CI [0.024, 0.501]). Significant differences were not found in the control group for either measure (Q15: *p* = 1, ϕ = 0.1; Q16: *p* = 1, ϕ = −0.1).

### 3.3. Cat Intervention Outcomes

#### 3.3.1. Cat–Child Attachment Styles (Secure Base Test)

McNemar tests were conducted on dichotomized attachment classifications (Secure vs. Insecure) at each timepoint. When evaluating the cat’s attachment style towards the child post-intervention/six weeks later compared to baseline (T1 to T2), no statistically significant change in attachment security was observed for either the CAT group (*n* = 17 dyads, *p* = 0.22, Cohen’s *g* = 0.33) or the control group (*n* = 15 dyads, *p* = 0.63, Cohen’s *g* = 0.25). However, long-term analysis comparing baseline to the one-year follow-up (T1 to T3) revealed a statistically significant increase in the number of cats with secure attachments towards their child partner in the CAT group (*n* = 15 dyads, *p* = 0.03, 95% CI not estimable due to zero-cell count, Cohen’s *g* = 0.50), with six cats moving from an insecure to a secure classification. No significant long-term change was observed in the control group over the same period (*n* = 11 dyads, *p* = 1.00, Cohen’s *g* = 0.00).

#### 3.3.2. Cat–Child Sociability

Wilcoxon Signed-Rank tests were conducted on proximity-seeking duration during Phase 1 and Phase 4. There was a statistically significant increase in this measure of cat sociability towards the child in the SBT Phase 4 (child-initiated) from T1 to T2 (*n* = 17, W = −58, *p* = 0.036 (one-tailed), rank-biserial correlation *r* = 0.48). There were no other significant effects on cat sociability, including during Phase 1 where the child remained passive (*p* > 0.05) and the significant effect was no longer detectable for the CAT group at T3 (*n* = 15, *W* = −20, *p* = 0.45, rank-biserial correlation *r* = 0.22).

#### 3.3.3. Program Acceptability and Engagement

Prior research has identified several key indicators of intervention acceptability; of these, session attendance has most consistently been associated with stronger program effects [37]. In the current study, program attendance was 100%, which is indicative of high intervention acceptability [34,38]. Every participant randomly assigned to the intervention (CAT) group attended all six training sessions. The study retention rate also surpassed expectations at 97.14% (i.e., beyond training session participation, 97.14% of participants also participated in research assessments at T1 and T2). Only one participant from the control group dropped out for an unknown reason prior to the second timepoint (T2). As expected, retention rates were lower at T3, given that this assessment was conducted one year past participation in the intervention. However, 77.8% of participants (28 of the 36 total participants) still returned for their 1-year follow-up assessments suggesting the strong potential of this intervention for long-term engagement.

A high mean homework completion rate of 81% indicated a strong level of participant engagement in the AAI program. This level of engagement is notable given that prior work suggests many students, particularly those with learning difficulties, may struggle to complete homework assignments (e.g., ~56% in some samples) [39]. Consistent with broader homework research highlighting the roles of task design and support in promoting completion and effort, several factors may have contributed to this high completion rate: the developmentally tailored, cat-focused nature of the assignments, their relative ease and limited time commitment, and the encouragement provided by trainers and guardians, which may have enhanced children’s motivation to engage in both the intervention activities and cat training at home [40,41].

### 3.4. Intervention Feasibility Results

#### Program Fidelity/Implementation

Fidelity scores ranged from 53.85% to 83.33% (*M* = 64.71%, *SD* = 16.70%, with the median Fidelity score for participants following the training protocol exactly as prescribed falling above the acceptable level cited in prior literature and established as the proposed benchmark for this study (>60%). The body language lesson, target stick training, and final training challenge demonstrate high adherence rates, exceeding 80%, while come and sit demonstrate low adherence rates, falling below 30%. Behavior completion scores ranged from 0 to 83.33% (*M* = 34.41%, *SD* = 29.56%). Fidelity and behavior completion showed a moderate positive correlation (*N* = 17, Spearman’s *p* = 0.41, *p* = 0.10), which was not statistically significant. These findings suggest that some protocol components may have been easier to implement or are more readily adopted by participants as designed, while others required greater flexibility. Additionally, Spearman’s rank correlation analysis detected no statistically significant association between cat age and behavior completion scores (*n* = 12, Spearman’s *p* = −0.48, *p* = 0.12; note that age data were unavailable for five participants), suggesting that age was not a predictive factor for training success within the limits of this sample size.

## 4. Discussion

This study aimed to evaluate the feasibility and impact of a novel Cat-Assisted Training (CAT) intervention designed to promote healthy behaviors in the form of joint activities and responsibility taking, and to promote emotional wellbeing by strengthening the bond between children with developmental disabilities and their family cats. A summary outcome analysis can be found in Supplemental Table S1.

### 4.1. Child–Cat Attachment

Limited research has focused on the child–cat bond, including within populations of children often recruited for AAI participation. Therefore, in the present study we felt that it was important to provide some preliminary data on the baseline child–cat relationship within a sample of children with developmental disabilities. This included an evaluation of the children’s caregiving roles, humane behavior towards animals, and feelings of attachment towards their own cat. Overall, children reported strong feelings of attachment towards their family cat. In fact, reported feelings of attachment were so strong in the population, even at baseline, that tracking improvements in this measure using the chosen instruments was challenging due to ceiling effects. This may suggest that other measures with more variability at baseline (such as the CCRI) may be especially important to include in future evaluations of AAI impact among populations that often demonstrate strong attachment to household animals. However, this finding did provide support for the idea that children can develop close bonds with cats, comparable to those reported towards dogs [42], and that cats can be a desirable or even preferred AAI partner for many children.

We also found that children who reported taking more responsibility for cat care and engaging more frequently in humane interactions such as play and cuddling at baseline also reported stronger General Attachment to their cats compared to those who did not. This is consistent with work linking children’s caring and friendship behaviors with higher pet attachment and more positive attitudes towards animals [43,44]. While the current findings cannot determine the directionality of this relationship, these findings suggest that a child’s attachment to their cat is strongly linked to their perceptions of their own role in caring for and interacting with the cat. The positive associations between cat care responsibility, humane behavior, and General Attachment are also consistent with attachment theory, which emphasizes the importance of active interaction and proximity in strengthening bonds [45], and with social-cognitive theory, which links caregiving experiences to self-efficacy, empathy, and an understanding of others’ needs [46]. By highlighting these dynamics in a population of children with developmental differences, this study offers insights into factors that may be important to supporting child–cat relationships at home or within AAI contexts that warrant additional study.

While cat attachment towards adult human caretakers has been demonstrated previously [10,28,47], this is also the first study to characterize attachment styles of cats towards a child in the home. This was accomplished using the SBT which measured the cat’s ability to use the child as a source of stress reduction and as a secure base (a bonded individual who facilitates stress resilience and exploration in a novel environment or context). The current study found that some cats did show behavior consistent with secure attachment towards their child partner at baseline (29% secure attachments overall at T1). However, as in prior studies exploring child-dog attachments, the rate of secure attachment to child partners was initially lower than what has been reported in studies of adult human-pet cat attachment (typically above 50% secure) [42], potentially due to children participating in less direct care for the animals compared with household adults as evaluated in the CCRI. This indicated both the potential for secure and supportive cat–child bonds from the cat’s perspective, and room for improvement that could be targeted within the intervention.

### 4.2. Intervention Impact

Overall, participation in the CAT intervention was associated with increases in reported child caregiving participation and specific indices of the cat–child relationship. Children in the CAT group showed marked increases in their reported participation in and perceived responsibility for cat care and increased engagement in shared activities. Similar to interaction-style interventions that enhance cats’ affiliative behavior toward humans by modifying human behavior [48], these findings suggest that structured training and caregiving support can rapidly shift child behavior in ways likely to be experienced as positive by cats. Significant increases in reported engagement in physically active behaviors such as walking and training the cat at home also increased in children participating in the CAT intervention, which echoes reported outcomes for dog-assisted interventions where walking is often treated as a meaningful health outcome for child participants, including participants with DD [49]. Cat training, including the current cat-training intervention, could provide the foundational skills (such as socialization and backpack/leash training) necessary for participants to engage in other joint activities with added health benefits. Future research on AAIs should continue to investigate if cats could be used as an alternative partner in walking-based health interventions, measuring physical activity more directly through activity or health monitors, and how health and wellbeing outcomes for human and animal participants compare to similar AAIs with dogs serving as the walking partner. Such research is important because cat walking interventions could provide greater accessibility to linked health benefits for segments of the population that do not have access to dogs, are afraid of dogs or have allergies, or could not safely handle a dog on leash (e.g., could be pulled down due to limited mobility or a dog’s larger size) but would be able to walk a cat.

We were also interested in outcomes related to cat behavior and wellbeing. At least one prior dog training AAI study demonstrated that participation in AAIs improved the dog-child bond, increasing rates of dog attachment security towards child partners [50]. In the current study we asked the same question about the cat–child bond. While at the six-week follow-up (T2), the CAT group did not yet show a significant increase in secure attachment rate towards their child partners, cats in the CAT group did show a significant increase in proximity seeking towards the child at T2 with no comparable change in controls. This is consistent with prior findings that cat sociability can be modulated fairly quickly by human attentional state and methods of human–animal interaction in the short term [48,51]. These data suggest that while children can quickly adopt new caregiving and interaction behaviors that have a more immediate effect on a cat’s motivation to approach the child (over the course of six weeks), a more extended period of time with the child implementing the behavioral changes learned in the intervention may be required to see a shift to the cat’s attachment style. This appears to be supported by the significant increase in the number of cats from the CAT intervention group showing attachment security towards their child partner one year later at T3 (while no such change was observed in controls). Given the constraints of pilot-scale attachment research and the limitations inherent in dichotomizing classification categories, these long-term findings should be interpreted with caution. They are best presented as preliminary indicators that warrant replication in future studies with greater statistical power. Nonetheless, our current findings are consistent with attachment theory, and prior findings, which point to attachment styles as relatively stable over time; with shifts typically only occurring after major life events, or longer-term consistent environmental or relationship changes [52]. Further research is needed to determine the specific mechanisms that drive such longitudinal shifts in feline attachment and to determine how long these positive changes in relationship dynamics might continue if evaluated more than a year post-intervention. However, these findings suggest it is possible to develop cat AAIs that lead to changes in interaction styles that may facilitate ongoing improvements to the child–cat relationship.

Importantly, this intervention demonstrates that it is possible to develop AAIs that consider both human and animal wellbeing, as well as the human–animal relationship. In addition to ensuring ethical and sustainable approaches to AAI, future research should evaluate the possibility that AAIs that promote improved animal wellbeing may facilitate more sustainable engagement and greater participant motivation. The current findings are also broadly consistent with theoretical and empirical work indicating that cat–human relationship quality may be shaped by both proximate interaction patterns, such as how and when humans attend to and handle their cats and longer-term environmental stability and caregiving history [48,53]. Future work should examine which specific caregiving behaviors (e.g., sensitivity to cat body language, respecting choice and control, consistency of routines) are most predictive of transitions to secure attachment, and whether such changes are sustained beyond one year.

### 4.3. Program Acceptability and Feasibility

The current study also demonstrated that an intervention focused on an active child–cat partnership, and more specifically a cat-training intervention, was highly desirable and acceptable to child participants and their families. We received more interest in the program during the recruitment period than the study was designed to enroll, resulting in a waitlist for future opportunities. For enrolled participants, the 100% session attendance rate and the 94% T1 to T2 assessment-completion retention rate far exceeded typical benchmarks for high acceptability [29,30], a critical indicator of the program’s feasibility in applied settings. Furthermore, 77% of participants were willing to come back to complete a final assessment one year after study initiation suggesting ongoing engagement and long-term commitment to the program. When considering these metrics together with intervention outcomes, the CAT intervention proved to be a feasible and accessible AAI program with measurable positive outcomes for both children and cats, that supported beneficial behaviors that youth with DD were highly motivated to engage in with their cat during and beyond the six in-person intervention sessions.

While more research is needed to determine what elements of the intervention might be most critical to achieving these outcomes, there are several elements of this intervention that seem like plausible contributors and warrant future investigation. First is the incorporation of a family pet in the intervention. Youth and families typically regard companion animals as close, trusted members of the household, and attachment to pets is linked with feelings of comfort, security, and emotional support across development [54,55]. In many children, strong pet bonds are also associated with better self-esteem, reduced loneliness, and broader social benefits, which can make pet-based activities feel inherently rewarding and worth sustained effort [56]. Animal-assisted intervention research further indicates that interactions with familiar animals can enhance motivation, engagement, and willingness to participate in therapeutic or educational tasks, partly because the animal serves as a salient, emotionally meaningful partner rather than a neutral tool [56]. In other words, mutually rewarding AAI partnerships with bonded pets may have a motivational advantage, as animals are a living reminder to engage in these activities and may prompt children for engagement in activities associated with positive outcomes for the animal (i.e., attention, treats, opportunities to explore/walk) beyond the intervention setting. Second, cat-training opportunities—especially those where youth can serve as the primary trainer—are still relatively novel. It is possible that the novel interventions, where children can learn unique skills or work with a species not as commonly included in AAIs, inspire greater engagement and long-term interest and motivation. Because children were also found to be strongly attached to their cats, it is possible that interventions that encourage children to take more of an active role in their cat’s care and wellbeing, improve child–cat relationships and result in their cat seeking their proximity for longer durations reinforce long-term engagement, especially for interventions incorporating a family cat that will continue to live in the home with the child. While further investigation into such sub-components of AAIs is valuable, it is also possible that a combination of relevant factors is necessary to achieve positive outcomes. Therefore, additional applied evaluation of integrated AAI approaches, such as the current evaluation of the CAT intervention, is also important and should be replicated in other populations and settings as a unit to further investigate the generalizability of the current findings.

### 4.4. Limitations

While the present study offers novel insights into the feasibility and potential relational impact of the CAT intervention, several constraints should be considered. Our modest sample size (*N* = 36 dyads) limits the statistical power of our analyses. As an initial feasibility study, our primary objective was to evaluate the intervention’s implementation and document preliminary indicators of change in the child–cat relationship. Several positive outcomes suggest that such interventions hold promise and warrant future study. However, it is also important to note that since this study was not powered to detect small effect sizes and because we also experienced some attrition over the course of the study, insignificant results reported here should be treated with caution and require further investigation with larger sample sizes. The study design also did not allow for participant blinding (as participants knew whether or not they were participating in a training class), which introduces the potential for expectancy effects and social desirability bias in self-reported outcomes in survey measures. While we also included some behavioral measures in this study, future studies should consider incorporating additional data on behavioral or physiological outcomes in child participants.

## 5. Conclusions

The current findings suggest that family cats have the potential to serve as active AAI partners in at least some settings. However, given the modest sample size and the exploratory nature of some of our findings, conclusions should be viewed as preliminary and requiring additional study. In general, more research aimed at novel interventions with family-cat partners has the potential to make AAIs more accessible by providing AAI access to those who cannot, or do not wish to, interact with common AAI species (such as dogs and horses) and by allowing participants to benefit from joint activities with an animal that is already living in their home. This study also provides evidence that it is possible for AAIs to be designed in ways that promote positive outcomes for both human and cat participants; more research on AAI methods aiming to improve both human and animal outcomes is needed. Finally, data suggesting high motivation for, and increases in, joint activities such as walking with cats post-intervention provide a promising foundation for future investigations exploring physical activity and health benefits of human–cat partnerships, a currently understudied area of research.

## Figures and Tables

**Table 1:** SBT Ethogram. Classification criteria for attachment styles in domestic cats [10].

Attachment Style	Definition
Secure	Cat’s greeting behavior is active, open, and positive. Little or no resistance to contact or interaction with the human participant. Seeks proximity and is comforted upon reunion, may return to exploration or play.
Insecure-Avoidant	Cat shows little or no visible response to the caretaker’s return. Ignores or turns away from caretaker but may not resist interaction altogether (e.g., laying, sitting, or standing without physical contact with, out of reach of, or at a distance from caretaker).
Insecure-Ambivalent	Cat shows exaggerated proximity-seeking and clinging behavior (but may struggle if held by caretaker). Exhibits a mix of persistent distress with efforts to maintain physical contact with the caretaker and/or physically intrusive behavior toward the caretaker. (Cats who the judges agreed seemed essentially secure but with ambivalent tendencies were categorized as secure).
Insecure-Disorganized	Cat exhibits evidence of a strong approach-avoidance conflict or fear upon reunion (e.g., circling caretaker, hiding from sight, rapidly dashing away upon reunion, or “aimless” wandering around the room). A lack of coherent strategy is shown by contradictory behavior. Cat may show stereotypies upon reunion (e.g., freezing or compulsive grooming). “Dissociation” may be observed, that is, still or frozen posture, staring into space without apparent cause, for at least 20 s (in a non-resting, non-sleeping cat).
Unclassified	Judges were unable to reach consensus on the attachment style categorization of the cat. Unclassifiable cats were excluded from further analysis on cat attachment.

**Table 2:** Baseline descriptive statistics for child-reported survey measures. (Source: Authors’ own research).

Measure	*N*	Mean (SD)	Response Range	Survey Range
LAPS-Total	35	0.68 (0.17)	0.28–0.96	0–1
LAPS-GA	35	0.79 (0.17)	0.25–1.00	0–1
CTAQ	36	29.5 (3.6)	22–36	13–39
CCRI	36	9.2 (3.4)	2–15	0–15

Note: LAPS and GA scores have been normalized to a 0–1 scale for analysis. Raw score ranges were 0–46 and 0–22, respectively.

**Table 3:** Baseline comparability between intervention and control groups.

	Intervention Group (Mean)	Control Group (Mean)	Test Statistic	*p*-Value
Cat Age (years)	3.34	3.55	Mann–Whitney Test	0.18
Cat Sex (% Female)	0.5	0.27	Fisher’s Exact Test	0.41
Cat Sex (% Male)	0.5	0.72	Fisher’s Exact Test	0.41
Child Age (years)	12.17	12.12	Mann–Whitney Test	0.63
Child Sex (% Female)	0.32	0.29	Fisher’s Exact Test	0.21
Child Sex (% Male)	0.68	0.53	Fisher’s Exact Test	0.21
Child Sex (% Other/Unknown)	0	0.18	Fisher’s Exact Test	0.21
T1 LAPS	0.66	0.65	Mann–Whitney Test	0.15
T1 CCRI	8.22	9.36	Mann–Whitney Test	0.33
T1 CTAQ	29.7	29.61	Mann–Whitney Test	0.051

Note: Data are presented as means for continuous variables (Age, LAPS, CCRI, CTAQ) and as proportions for categorical variables (Sex).

## Data Availability

Raw group-level deidentified data supporting the conclusions of this article will be made available by the authors on request in a manner permissible by the associated IRB.

## Supplementary Materials

The following supporting information can be downloaded at: https://www.mdpi.com/article/10.3390/ani16142133/s1, Table S1: Protocol component adherence across sessions. (Source: Authors’ own research). The results revealed high variability in protocol adherence rates during different parts of the cat-training classes (when training different behaviors). The body language lesson, target stick training, and final training challenge demonstrate high adherence rates, exceeding 80%, while come and sit demonstrate low adherence rates falling below 30%. These findings suggest that some protocol components may be easier to implement or are more readily adopted by participants as designed, while others required greater flexibility.
