# Supplementary material for: Evaluating a Novel Cat-Assisted Training (CAT) Intervention for Youth with Developmental Disabilities and Their Family Cat
Source: Animals (Basel). 2026 Jul 9;16(14):2133. doi: 10.3390/ani16142133 (PMC13404458; doi:10.3390/ani16142133)
Supplement: Supplementary file 1 [file animals-16-02133-s001.zip › animals-4416433-supplementary.pdf]

Supplementary Materials

Table S1: Protocol component adherence across sessions. (Source: Authors' own research). The results revealed high variability in protocol adherence rates during different parts of the cat training classes (when training different behaviors). The body language lesson, target stick training, and final training challenge demonstrate high adherence rates, exceeding 80%, while come and sit demonstrate low adherence rates falling below 30%. These findings suggest that some protocol components may be easier to implement or are more readily adopted by participants as designed, while others required greater flexibility.

Session	Protocol Component	Participant Adherence Proportion
1	Body Language Lesson	0.89
	Harness Backpack Intro	0.67
2	Training Mechanics	0.56
	Target Stick Intro	0.78
	Name Attention	0.5
3	Watch Me	0.56
	Come	0.29
	Target Stick Cont.	0.83
4	Sit	0.28
	Target Stick Cont.	0.78
5	Go To Mat	0.5
	Target Stick Cont.	0.94
6	Training Challenge	0.83
